# A Versatile Ultra-High-Performance Liquid Chromatography-Full-Scan High-Resolution Mass Spectrometry Method to Quantify Wine Polyphenols

**DOI:** 10.3390/mps7050082

**Published:** 2024-10-10

**Authors:** Damien Flores, Emmanuelle Meudec, Aécio Luís de Sousa Dias, Nicolas Sommerer

**Affiliations:** 1 SPO, Université de Montpellier, F-34000 Montpellier, Franceaecio-luis.de-sousa-dias@inrae.fr (A.L.d.S.D.); 2 INRAE, Institut Agro, F-34000 Montpellier, France; 3 INRAE, PROBE Research Infrastructure, PFP Polyphenol Analysis Facility, F-34060 Montpellier, France

**Keywords:** UHPLC-HRMS, Orbitrap mass spectrometry, anthocyanins, flavonoids, flavonols, tannins

## Abstract

Polyphenols are responsible for wine colour and astringency, and, as antioxidants, they also have beneficial health properties. In this work, we developed a robust full-scan high-resolution mass spectrometry method for the quantification of 90 phenolic compounds in wine samples (either red, rosé, or white wine), using a UHPLC-Orbitrap^TM^ system. With this method, we could conduct a detailed analysis of phenolic compounds in red, rosé, and white wines with great selectivity due to sub-ppm mass accuracy. Moreover, accessing the full-scan spectrum enabled us to monitor all the other compounds detected in the sample, facilitating the adaptability of this method to new phenolic compounds if needed.

## 1. Introduction

Phenolic compounds play an important role in wine quality, as they are responsible for certain organoleptic qualities such as colour, astringency, and bitterness [1]. They are also known for their health benefits, when consumed in moderation—for instance, their antioxidant and antimicrobial properties or their ability to prevent or reduce the progression of diseases including cancer, cardiovascular disease, and Alzheimer’s disease [2]. Phenolic compounds are suggested as chemical markers for the authentication and varietal differentiation of grapes and wines [3]. Researchers have suggested that flavonol profiles could be used as a general chemical indicator for the authenticity of both red and white *Vitis vinifera* grape cultivars and their corresponding single-cultivar wines [4]. The relative proportions of individual anthocyanin components are typical for each grape variety and range within specific defined limits [5], and these compounds can be used to authenticate the grape variety [6].

The characterization and quantification of phenolic compounds in wine is usually carried out by liquid chromatography with a UV–visible detector (LC-UV) or liquid chromatography coupled with mass spectrometry (LC-MS) [7]. More recently, analytical methods based on ultra-high-performance liquid chromatography coupled with a triple quadrupole mass spectrometer (UHPLC-QqQ-MS) using the Multiple Reaction Monitoring (MRM)/Selected Reaction Monitoring (SRM) detection mode were developed in order to increase selectivity and sensitivity while reducing analysis time [8,9]. With this technique, it has been possible to quantify 152 wine polyphenols within a 30 min UHPLC gradient, without sample pre-treatment apart from wine filtering [8]. However, a drawback of this method is that compounds are targeted, and adding or removing molecules changes the dwell time of the other targeted ions and modifies the method performance and accuracy. With the advent of high-resolution mass spectrometry (HRMS), we have seen the emergence of new techniques for targeted quantitative approaches as new acquisition modes for acquiring high-resolution full-scan spectra and MS/MS spectra at the same time [10,11]. Access to the full-scan spectrum allows us to monitor, if later needed, all the compounds detected in the sample. It is then very easy to add or remove compounds for quantification without modifying the method and method validation. Thanks to the broad dynamic range in the full scan acquisition mode of the Orbitrap^TM^ mass analyser, together with a flexible and easily adaptable method, we can achieve sensitive analysis of wine polyphenols. MS/MS data are then acquired solely to assess compound identification, based on characteristic fragment ions. The aim of the work reported here is to present a new sensitive and selective method for quantifying, in full-scan high-resolution mass spectra, 90 phenolic compounds. The data acquisition was performed on an ultra-high-performance liquid chromatography system coupled to a high-resolution Orbitrap^TM^ mass spectrometer. It is noteworthy that, thanks to its versatility, this method can be used to quantify polyphenols from red, rosé, or white wines, not considering the anthocyanins in white wines.

## 2. Materials and Methods

Formic acid and HPLC-grade acetonitrile were purchased from Biosolve (Dieuze, France). The water was of Milli-Q quality (Milli-Q purification system, Millipore, Molsheim, France). Gallic acid, caffeic acid, and ethyl protocatechuate were purchased from Sigma Aldrich (Saint-Louis, MO, USA). Ferulic acid was purchased from Honeywell Fluka (Buchs, Switzerland). *Trans*-caftaric acid was purchased from Phytolab (Vestenbergsgreuth, Germany). Standards of p-coumaric acid, protocatechuic acid, vanillic acid, ethyl caffeate, syringic acid, procyanidin B1, procyanidin B2, procyanidin B3, procyanidin A2, procyanidin C1, malvidin 3-*O*-glucoside chloride, malvidin 3,5-*O*-diglucoside chloride, *trans*-piceid, *trans*-resveratrol, *trans*-ε-viniferin, (+)-catechin, (−)-epicatechin, quercetin dihydrate, and quercetin 3-*O*-glucoside were purchased from Extrasynthese (Geney, France). The Pech Rouge experimental unit vineyard (UE-PR, INRAE, Gruissan, France) supplied the four single-varietal red wine samples used to validate the method (grape varieties: Jacquez, Artaban, Merlot and Pinot Noir).

UHPLC-HRMS analysis were performed using a Vanquish Flex system (Thermo Fisher Scientific, Bremen, Germany) hyphenated to an Orbitrap^TM^ Exploris 480 (Thermo Fisher Scientific, Bremen, Germany) equipped with a heated electrospray source (H-ESI) working in the positive or negative mode. The UHPLC system includes a thermostatic autosampler (10 °C) and a thermostatic column compartment (35 °C). Chromatographic separation was performed on a reverse-phase Acquity HSS T3 column 1.8 µm 1.0 mm × 100 mm (Waters, Saint-Quentin-en-Yvelines, France). The mobile phases consisted of phase A, water/formic acid (99:1, *v*/*v*), and phase B, acetonitrile/water/formic acid (79.5:19.5:1, *v*/*v*/*v*). The flow rate was 0.22 mL/min, and the elution gradient was as follows: 2% solvent B for 1.5 min, 2% solvent B to 12% solvent B in 3 min, 12% solvent B for 2.5 min, 12% solvent B to 24% solvent B in 5 min, 24% solvent B to 48% solvent B in 3 min, 48% solvent B to 60% solvent B in 1 min, 60% solvent B to 100% solvent B in 1 min, 100% solvent B for 2 min, and 100% solvent B to 2% solvent B in 1 min, followed by the washing and reconditioning of the column (4 min, 2% B). Injection volume was 0.5 µL.

H-ESI conditions were as follows: vaporizer temperature, 300 °C; sheath gas, 40 arbitrary units (au); sweep gas, 2 au; spray voltage, 3500 V in positive mode and 2500 V in negative mode; ion transfer tube temperature, 280 °C; scan range, 150–1200 *m*/*z*; intensity threshold, 5.0 × 10^4^; and MS full-scan resolution, 120 K at *m*/*z* 200. The MS/MS parameters were as follows: resolution, 30 K; normalized HCD (Higher-energy Collisional Dissociation) collision energy of 35%. The TraceFinder 4.1 software (Thermo Fisher Scientific) was used to process the data. The instrument performed data-dependent acquisitions using an inclusion list containing the *m*/*z* values of the targeted compounds. In this acquisition mode, the ions corresponding to the *m*/*z* values present on the inclusion list and with an intensity above the aforementioned intensity threshold were selected one at a time to be fragmented in MS/MS experiments. The inclusion list facilitated the collection of diagnostic MS/MS spectra for the verification of the targeted compounds. Indeed, the resulting MS/MS spectra were used for the annotation process and for analyte identity confirmation (i.e., “qualifier ion”).

The metabolite identification was based on the work of Schymanski et al. [12], where four levels of metabolite identification were proposed. The compounds were annotated based on the retention times, predicted molecular formulae, and MS/MS fragmentation patterns that were compared with the available standards and with our internal database and the literature.

Sub-ppm mass accuracy is ensured throughout the UHPLC-HRMS acquisition by the Easy-IC^TM^ feature of the Exploris Orbitrap Mass Spectrometer. This technology introduces a controlled number of internal calibrant ions, enabling the instrument to continuously adjust the mass-to-charge ratio calibration in real time. This process corrects for errors that would otherwise remain uncorrected due to temperature fluctuations and variations in the total ion population between scans.

## 3. Results

### 3.1. Preliminary Parameters

The phenolic compounds targeted in this method resulted in level 1 (when standards were available) and level 2 identifications [12]. They were chosen after a preliminary non-targeted analysis in order to reduce the list of molecules already used previously for the analysis of rosé wines [8]. The selection was made by choosing the compounds that allowed us to discriminate not only between different grape varieties but also between compounds present in sufficiently abundant quantities to be detected and quantified. This analysis provided us with the exact *m*/*z* (to within 1 ppm), retention time, and *m*/*z* of the MS/MS fragments of each compound, as shown in Table 1.

### 3.2. Linearity and Limits of Detection and Quantification

The linearity of the method was evaluated using standard solutions at seven concentration levels (0.01 to 500 µmol/L) for the 24 compounds concerned. This range was too wide; the signal was saturated at 500 µmol/L and was too weak to be accurately quantified at 0.01 µmol/L, so it was reduced to five concentration levels, from 1 to 250 µmol/L. The accuracy values for the different concentration levels of the calibration curves are presented in Table S1 of the Supplementary Material. These values were converted into mg/L in Table 2 to make them more informative. We obtained linear regression coefficients R^2^ greater than 0.998 for 22 compounds, except for p-coumaric acid (0.995) and *trans*-piceid (0.994). Focusing on the equations of the calibration curves, we noticed that the y-intercepts of the Malvidin 3,5-*O*-diGlc and Malvidin 3-*O*-Glc equations were too high (1.2 × 10^7^ and 6.53 × 10^7^ respectively) to be able to quantify the other compounds associated with these two standards. We therefore decided to force the curve to pass through the origin, in order to avoid the y-intercept, and we could quantify the compounds whose areas were less than these y-intercept values. We noted that, with these modifications, the slope values did not vary significantly (0.4% for Malvidin 3,5-*O*-diGlc and 1.3% for Malvidin 3-*O*-Glc), neither did the concentrations obtained using the equations with and without the y-intercept (0.4% difference for Malvidin 3,5-diGlc and 3.8% for Malvidin 3-*O*-Glc).

The detection limit (LOD) for each compound was calculated, as shown in Table 2, using the method presented by Miller and Miller [13], based on the equation for the linear regression curve. LOD values calculated with this method were in the range of 0.002–0.061 mg/L. Our quantification limits (LOQs) were set at 1 µmol/L for all compounds, as this was the lowest point in our calibration range, and we did not quantify below this concentration.

Quantification was carried out by integrating the peak corresponding to the precursor ion; the MS/MS fragments were only used to confirm the allocation of the peak to the correct compound. A minimum of 10 data points per peak was set for quantification.

Given that we had a wide range of compounds to quantify, many of them were not commercially available, so we could not make an absolute quantification on the compounds concerned; therefore, we quantified them by equivalence with reference standards of the same molecular class. For all the method validation tests, the wine analysed was an equivolumic mixture of the four selected wines, made after centrifuging the wine at 18,000 rpm for 15 min and diluting by four with a mixture of water, methanol, and formic acid (49.5/49.5/1 *v*/*v*/*v*).

### 3.3. Repeatability and Inter-Day Precision

According to the AFNOR NF V03-110 standard method, titled “Protocole de caractérisation en vue de la validation d’une méthode d’analyse quantitative par construction du profil d’exactitude”, based on the work of Hubert et al. [14] to calculate repeatability and inter-day precision, it is specified that three repetitions at three different concentration levels should be performed over three days. Since the standards were not available for all of the compounds, we decided to use our wine sample at three different dilutions (1:3, 1:5, 1:7 *v*/*v*, noted as Dil. 1:3, Dil. 1:5, and Dil. 1:7, respectively, in Table 2). The dilution solvent was a mix of methanol, water, and formic acid (49.5/49.5/1 *v*/*v*/*v*).

We analyzed our samples in sets of 10 repetitions per day for each of the three dilution levels, over three consecutive days, and for the two ionization modes. Ionization source cleaning and Orbitrap^TM^ mass analyser calibration were conducted between each day of analysis.

Regarding repeatability, we extracted the area of each peak and calculated the mean and the standard deviation of the areas for each compound from the 10 repetitions for each dilution level and for the first day of injection. We expressed the result as the Coefficient of Variation (CV %), which corresponds to the ratio of the standard deviation to the mean (Relative Standard Deviation).

For inter-day precision, we relied on repetitions over 3 consecutive days, calculating the average of the areas for each dilution level over the 3 days and the inter-day precision standard deviation. Details of the calculations can be found in Hubert et al. [14]. However, we adapted the calculation method present in these works by replacing concentration values with area values due to the unavailability of the standards. We also expressed the result as a coefficient of variation in percentage, as shown in Table 2.

The repeatability studies were acceptable, with coefficients of variation below 15% for all compounds except six. However, we noted that these six compounds were present in smaller quantities, which explains the significant variations between samples.

We noted similar results for inter-day precision, with coefficients of variation below 25%, except for seven compounds.

### 3.4. Matrix Effects

As with the previous tests, we did not have all the standards available for our compounds. To calculate or estimate the matrix effect, we could not rely on the calculation of the ratio between the peak area of analytes recorded for the sample spiked with standards and the peak area of analytes recorded for the standard solution (expressed as a percentage). We decided to inject our wine blend at five different dilutions (1:1; 1:3; 1:9; 1:19; 1:49) and inject our wine blend undiluted. We then integrated each peak and plotted the graph representing the area against the dilution factor. If there was no matrix effect, we would have a linear response. We observed that, for all compounds, the response was linear, from a 2-fold dilution up to a 20-fold dilution. As a precaution, we decided to dilute our wine samples by four to avoid losing certain compounds due to excessive dilution, to mitigate matrix effects, and to prevent instrument saturation.

### 3.5. Acquisition Parameters

Our injection sequence was composed as follows: two blanks (water) to stabilize the column with the elution gradient. Next, we injected the calibration solutions, from the least concentrated to the most concentrated. We followed with the core of the sequence composed of a blank injection and then one sample injected in triplicate. We repeated this core of sequence according to the number of samples to analyze. For long sequences (more than 50 samples), we reinjected the calibration range at the end to assess signal stability throughout the injection sequence. In this way, we observed good stability of the spectral signals. Even so, an analysis of a QC sample every 10 injections could be performed for long sequences to monitor the analytical responses.

## 4. Discussion

The novelty of this method lies in the fact that we quantified wine polyphenols directly from the full-scan MS signal, unlike SRM/MRM quantification methods, where quantification is performed on the fragment ion. The innovation and advantages of this method are its simplicity, speed, and adaptability. Selectivity is based on retention time, accurate (sub-ppm) mass measurements assessing the molecular formulas, and one or two qualifier fragment ions (for confirmation purposes only). The sensitivity is high due to high signal intensity on the full-scan MS (without signal losses due to precursor ion selection and MS/MS fragmentation) and a low noise level based on the analyzer’s high dynamic range.

The main appeal of this method is its versatility, with no need to revalidate the whole method when adding or removing new compounds to the quantification list, as the full-scan signal remains identical. Conversely, dedicated SRM/MRM triple quadrupole quantitation values are modified, either altered when adding new compounds or improved when removing compounds. This is intrinsically due to changes in dwell times in the quadrupoles for compounds overlapping or nearly overlapping during chromatography (i.e., multiple transitions monitored within the same time window). This method has been adapted to quantify wine polyphenols in red, rosé, and white wines, including when mixed in the same sample set. In the last case of white wines, anthocyanin compound series should not just be considered for data treatment, thus reducing the time for data reviewing.

Furthermore, for small- or medium-sized laboratories, one important point of interest of this method is its applicability on a single instrument (i.e., the Exploris mass spectrometer series) that is suited for both identification and quantification, with the same laboratory crew trained on a single versatile instrument and software suite. Moreover, to further increase laboratory throughput on wine polyphenol quantitation, a reduced list of compounds to monitor can be set for fast data reviewing. If needed after this data review, additional compounds may be monitored afterwards without new experimental acquisition, as the full-scan signal and, thus, the information are already available. This may be the case for a limited number of scientific questions, e.g., in monitoring anthocyanin derivative levels in red wines made from non-conventional grape varieties. In such a case, and for a reduced number of wine samples, it may be appropriate to monitor the less abundant pyranopeonidin and pyranopetunidin acetyl, coumaroyl, carboxy-acetyl, and carboxy-coumaroyl derivatives. Those derivatives are only monitored, as the first overview in a complete set of samples, for the more intense pyranomalvidin ion series.

When comparing with the data obtained in quantifying wine polyphenols with our previous MRM method on a standard triple quadrupole instrument [8], we did not notice a detrimental loss of accuracy. To demonstrate that our method achieved results comparable to others works (Myrtsi et al. [7]), but with greater versatility, two tables are provided (Supplementary Data Tables S2 and S3). This comparison is purely informational, as the datasets are not identical.

Supplementary Table S2 shows the LOD values of the present method compared with the LOD values of a triple quadrupole LC–MS/MS method developed by Myrtsi et al. [7]. The present method achieves better performance for the compared compounds, except for Malvidin 3-*O*-Glc, which has a LOD of 0.04 mg/L, while the other study reported a value of 0.02 mg/L. These results demonstrate that our UHPLC-HRMS provides good sensitivity for the studied compounds. This is useful for untargeted analyses aimed at comparing samples based on a large number of metabolites.

The concentration ranges for several compounds were also compared with the results of two MRM methods using triple quadrupole instruments in Supplementary Table S3. This table shows that, in general, our method is able to quantify the compounds when they are in higher concentrations compared to the other methods. The concentration ranges of the present method are appropriate, as they include the typical concentrations observed in wines. Furthermore, low-abundance compounds have, in most cases, a minimal impact on the organoleptic properties of wine and are less commonly used as quality markers. Therefore, the proposed method is well suited for the evaluation of wine quality, even with relatively higher concentration ranges. Indeed, this method is useful to analyze large panels of wine samples, enabling to determine whether the phenolic profile of these samples can be linked to different parameters such as origins, vintages, varieties, drought or environmental status, and the wine-making process.

Finally, the present method uses one ionization mode (either positive or negative) at a time, whereas in triple quadrupole methods, it is possible to use both modes in the same analysis. However, it is worth also noting that only nine compounds (phenolic acids) were analyzed in the negative mode, while the remaining compounds (anthocyanins and other flavonoids) were analyzed in the positive mode. Therefore, the analysis time of the present method can be reduced by half if phenolic acids are not the focus of a study, as, in this case, the samples would only need to be analyzed in the positive mode.

## Figures and Tables

**Table 1:** List of quantified phenolic compounds and MS parameters (ion mode, retention times, precursor, and fragments *m*/*z*).

Compound Family	Compound	Ion Mode (Negative (−) or Positive (+))	RT (min)	Precursor Ion (*m*/*z*)	MS/MS Fragments
Benzoic acids and derivatives	Gallic acid	−	1.02	169.0142	125.0245; 79.0190
	Protocatechuic acid	−	1.89	195.0652	109.0295
	Syringic acid	+	7.57	197.0455	169.0142; 124.0166
	Vanillic acid	+	5.59	169.0495	125.0598; 93.0335
	Ethyl protocatechuate	−	11.47	181.0507	153.0193; 108.0216
Hydroxycinnamic acids and derivatives	Caffeic acid	−	5.98	179.0349	135.0452; 107.0503
	p-coumaric acid	−	7.80	163.0395	119.0503
	Ferulic acid	+	9.31	195.0652	178.0580
	*trans*-caftaric acid	−	4.20	311.0408	179.0350; 149.0091
	Fertaric acid	-	6.79	325.0565	193.0506; 134.0373
	GRP (*cis*- and *trans*-isomers)	−	5.06; 5.70	616.1087	149.0092; 167.0172
	Ethyl caffeate	−	15.28	207.0663	179.0350; 134.0374
Stilbenes	*trans*-piceid	+	10.82	390.1310	229.0780
	*trans*-resveratrol	+	13.70	229.0800	135.0440
	(+)-ε-Viniferin	+	16.35	455.1489	361.1074
Flavonols	Quercetin 3-*O*-Glc ^a^	+	12.77	465.1028	303.0501; 85.0284
	Quercetin 3-*O*-glucuronide	+	12.55	479.0821	303.0500; 113.0233
	Quercetin	+	15.69	303.0499	190.8728
	Myricetin 3-*O*-Glc	+	10.93	481.0978	319.0452; 85.0284
	Myricetin 3-*O*-glucuronide	+	10.78	495.0769	332.0849; 319.0451
	Laricitrin 3-*O*-Glc	+	12.96	495.1133	333.0610; 97.0284
	Kaempferol 3-*O*-Glc	+	13.86	449.1080	287.0551; 85.0284
	Isorhamnetin 3-*O*-Glc	+	14.26	479.1185	317.0659; 85.0284
	Syringetin 3-*O*-Glc	+	14.39	509.1290	347.0763; 85.0284
Flavan-3-ols	Procyanidin C1	+	8.83	867.2122	407.0764; 245.0444
	Procyanidin A2	+	11.96	577.1342	287.0550
	Procyanidin B1 + Procyanidin B3	+	5.92	579.1499	287.0552; 409.0924
	Procyanidin B2	+	6.92	579.1500	287.0551; 409.0927
	Procyanidin B4	+	7.35	579.1498	287.0551; 409.0920
	Catechin	+	5.45	291.0863	139.0390; 123.0441
	Epicatechin	+	7.36	291.0863	139.0390; 123.0441
	Galloylated procyanidin dimer	+	9.79	731.1609	127.0390; 139.0390
Anthocyanins	Malvidin 3,5-*O*-diGlc	+	7.97	655.1876	331.0816; 493.1345
	Delphinidin 3,5-*O*-diGlc	+	6.14	627.1561	303.0501; 465.1035
	Cyanidin 3,5-*O*-diGlc	+	6.62	611.1611	287.0552; 449.1083
	Petunidin 3,5-*O*-diGlc	+	6.91	641.1717	317.0658; 479.1191
	Peonidin 3,5-*O*-Glc	+	7.69	625.1766	301.0708; 463.1237
	Malvidin 3-*O*-Glc	+	11.33	493.1343	331.0816; 315.0511
	Delphinidin 3-*O*-Glc	+	7.36	465.1028	303.0501
	Cyanidin 3-*O*-Glc	+	8.41	449.1077	287.0551
	Petunidin 3-*O*-Glc	+	9.61	479.1185	317.0659; 302.0424
	Peonidin 3-*O*-Glc	+	10.79	463.1237	301.0709; 286.0478
	Delphinidin 3-*O*-acetyl-Glc	+	12.32	507.1133	303.0501
	Cyanidin 3-*O*-acetyl-Glc	+	13.11	491.1185	287.0550
	Petunidin 3-*O*-acetyl-Glc	+	13.47	521.1289	317.0659; 202.0896
	Peonidin 3-*O*-acetyl-Glc	+	14.22	505.1342	301.0708; 286.0476
	Malvidin 3-*O*-acetyl-Glc	+	14.41	535.1449	331.0815; 315.0522
	Malvidin 3-*O*-acetyl-Glc-5-O-Glc	+	11.68	697.1977	331.0815; 535.1452
	Delphinidin 3-*O*-coumaroyl-Glc (*cis*- and *trans*-isomers)	+	13.86; 14.53	611.1398	303.0501; 147.0440
	Cyanidin 3-*O*-coumaroyl-Glc (*cis*- and *trans*-isomers)	+	14.46; 14.87	595.1448	287.0553
	Petunidin 3-*O*-coumaroyl-Glc (*cis*- and *trans*-isomers)	+	14.76; 15.00	625.1554	317.0659; 302.0423
	Peonidin 3-*O*-coumaroyl-Glc (*cis*- and *trans*-isomers)	+	15.07; 15.31	609.1605	301.0708; 286.0474
	Malvidin 3-*O*-coumaroyl-Glc (*cis*- and *trans*-isomers)	+	15.09; 15.36	639.1714	331.0816; 315.0515
	Delphinidin 3-*O*-coumaroyl-Glc-5-*O*-Glc (*cis*- and *trans*-isomers)	+	12.55; 13.04	773.1929	303.0501; 147.0442
	Cyanidin 3-*O*-coumaroyl-Glc-5-O-Glc (*cis*- and *trans*-isomers)	+	13.41; 13.73	757.76	287.0551; 147.0443
	Petunidin 3-*O*-coumaroyl-Glc-5-O-Glc (*cis*- and *trans*-isomers)	+	13.18; 13.83	787.2084	317.0659; 625.1597
	Peonidin 3-*O*-coumaroyl-Glc-5-O-Glc (*cis*- and *trans*-isomers)	+	13.89; 14.47	771.2127	301.0708; 609.1592
	Malvidin 3-*O*-coumaroyl-Glc-5-*O*-Glc (*cis*- and *trans*-isomers)	+	13.99; 14.47	801.2241	331.0817; 639.1715
	(epi)cat-ethyl-malvidin 3-*O*-Glc (4 isomers)	+	13.29; 13.57; 13.89; 14.16	809.2291	357.0973; 495.1287
	(epi)cat ^b^-ethyl-malvidin 3-*O*-acetyl-Glc (2 isomers)	+	14.94; 15.27	851.2385	357.0972; 539.12
	(epi)cat-ethyl-malvidin 3-*O*-coumaroyl-Glc (2 isomers)	+	15.31; 15.80	955.2649	357.09; 331.0814
	Pyranopeonidin 3-*O*-Glc	+	12.40	487.0857	325.0324
	Pyranopetunidin 3-*O*-Glc	+	10.96	503.1176	341.0659
	Pyranomalvidin 3-*O*-Glc (vitisin B)	+	12.54	517.1338	355.0818; 339.0500
	Pyranomalvidin 3-*O*-acetyl-Glc	+	13.36	559.1446	355.0815; 339.0493
	Pyranomalvidin 3-*O*-coumaroyl-Glc	+	15.00	663.1710	355.0817; 340.0582
	Carboxypyranomalvidin 3-*O*-Glc (vitisin A)	+	12.02	561.1238	399.0713; 383.0415
	Carboxypyranomalvidin 3-*O*-acetyl-Glc	+	12.68	603.1343	399.0713; 383.0411
	Carboxypyranomalvidin 3-*O*-coumaroyl-Glc	+	14.49	707.1608	399.0714; 383.0431
	Petunidin 3-*O*-caffeoyl Glc	+	14.15	641.1504	317.0659
	Peonidin 3-*O*-caffeoyl Glc	+	14.88	625.1552	301.0708
	Malvidin 3-*O*-caffeoyl Glc	+	14.75	655.1647	331.0816

^a^ Glc: glucoside; ^b^ epi(cat): catechin or epicatechin.

**Table 2:** Method validation parameters: correlation coefficients (R^2^), limits of detection (LOD), calibration ranges, repeatability, and inter-day precision.

Compounds	R^2^	LOD (mg/L)	Calibration Range	Repeatability (%)			Inter-Day Precision (%)		
			(mg/L)	Dil. 1:3	Dil. 1:5	Dil. 1:7	Dil. 1:3	Dil. 1:5	Dil. 1:7
Gallic acid	0.999	0.003	0.17–42.5	5	3	5	13	7	9
Protocatechuic acid	0.999	0.003	0.15–38.5	5	4	5	7	7	9
Syringic acid	0.999	0.003	0.20–49.5	3	2	3	13	5	7
Vanillic acid			0.17–42.0	18	10	14	21	15	28
Ethyl protocatechuate	1.000	0.002	0.18–45.5	3	4	6	15	12	13
Caffeic acid	0.999	0.011	0.18–45.0	2	1	1	12	4	6
p-coumaric acid	0.995	0.023	0.16–41.0	11	8	15	19	12	22
Ferulic acid	0.999	0.005	0.19–48.5	nd ^d^	nd	nd	nd	nd	nd
*trans*-caftaric acid	1.00	0.003	0.31–78.1	3	2	3	13	5	7
Fertaric acid	NA ^c^	NA	As equivalents of *trans*-caftaric acid	3	2	3	14	7	10
GRP (*cis*- and *trans*-isomers)	NA	NA	As equivalents of *trans*-caftaric acid	5	3	3	21	13	18
Ethyl caffeate	0.999	0.005	0.21–52.1	5	4	4	16	10	13
*trans*-piceid	0.994	0.061	0.39–97.6	nd	nd	nd	nd	nd	nd
*trans*-resveratrol	0.999	0.011	0.23–57.1	6	8	4	28	28	30
(+)-ε-Viniferin	0.999	0.025	0.45–113.6	15	23	16	17	21	20
Quercetin 3-*O*-Glc ^a^	0.999	0.006	0.46–116.1	5	2	4	14	11	14
Quercetin 3-*O*-glucuronide	NA	NA	As equivalents of Quercetin 3-*O*-Glc	5	1	1	19	14	19
Quercetin	0.999	0.024	0.30–75.6	4	3	3	19	16	20
Myricetin 3-*O*-Glc	NA	NA	As equivalents of Quercetin 3-*O*-Glc	4	2	1	16	14	18
Myricetin 3-*O*-glucuronide	NA	NA	As equivalents of Quercetin 3-*O*-Glc	7	2	19	19	14	17
Laricitrin 3-*O*-Glc	NA	NA	As equivalents of Quercetin 3-*O*-Glc	5	2	2	16	13	18
Kaempferol 3-*O*-Glc	NA	NA	As equivalents of Quercetin 3-*O*-Glc	5	3	4	18	14	19
Isorhamnetin 3-*O*-Glc	NA	NA	As equivalents of Quercetin 3-*O*-Glc	4	5	4	15	10	13
Syringetin 3-*O*-Glc	NA	NA	As equivalents of Quercetin 3-*O*-Glc	6	2	2	18	14	20
Procyanidin C1	0.999	0.020	0.87–216.7	8	3	3	32	24	23
Procyanidin A2	0.999	0.023	0.58–144.1	nd	nd	nd	nd	nd	nd
Procyanidin B1 + Procyanidin B3	0.999	0.004	0.58–144.1	6	2	2	17	9	14
Procyanidin B2	0.999	0.011	0.58–144.1	6	3	2	20	13	17
Procyanidin B4	NA	NA	As equivalents of Procyanidin B2	7	3	2	21	13	17
Catechin	0.999	0.011	0.29–72.6	4	4	4	12	7	11
Epicatechin	0.999	0.012	0.29–72.6	6	3	3	14	8	12
Galloylated procyanidin dimer	NA	NA	As equivalents of Epicatechin	6	2	2	23	16	19
Malvidin 3,5-*O*-diGlc	0.999	0.026	0.66–163.8	4	6	7	12	11	15
Delphinidin 3,5-*O*-diGlc	NA	NA	As equivalents of Malvidin 3,5-*O*-diGlc	7	5	6	9	13	13
Cyanidin 3,5-*O*-diGlc	NA	NA	As equivalents of Malvidin 3,5-*O*-diGlc	4	11	7	8	13	16
Petunidin 3,5-*O*-diGlc	NA	NA	As equivalents of Malvidin 3,5-*O*-diGlc	3	7	6	13	9	9
Peonidin 3,5-*O*-diGlc	NA	NA	As equivalents of Malvidin 3,5-*O*-diGlc	2	7	3	14	10	7
Malvidin 3-*O*-Glc	0.998	0.040	0.49–123.4	5	11	5	13	16	10
Delphinidin 3-*O*-Glc	NA	NA	As equivalents of Malvidin 3-*O*-Glc	3	8	8	10	15	13
Cyanidin 3-*O*-Glc	NA	NA	As equivalents of Malvidin 3-*O*-Glc	8	12	8	14	20	13
Petunidin 3-*O*-Glc	NA	NA	As equivalents of Malvidin 3-*O*-Glc	6	9	7	22	13	22
Peonidin 3-*O*-Glc	NA	NA	As equivalents of Malvidin 3-*O*-Glc	6	12	14	14	16	18
Delphinidin 3-*O*-acetyl-Glc	NA	NA	As equivalents of Malvidin 3-*O*-Glc	5	9	6	12	22	16
Cyanidin 3-*O*-acetyl-Glc	NA	NA	As equivalents of Malvidin 3-*O*-Glc	5	13	9	13	22	22
Petunidin 3-*O*-acetyl-Glc	NA	NA	As equivalents of Malvidin 3-*O*-Glc	6	9	6	14	20	21
Peonidin 3-*O*-acetyl-Glc	NA	NA	As equivalents of Malvidin 3-*O*-Glc	8	12	16	16	18	14
Malvidin 3-*O*-acetyl-Glc	NA	NA	As equivalents of Malvidin 3-*O*-Glc	7	11	5	15	16	8
Malvidin 3-*O*-acetyl-Glc-5-Glc	NA	NA	As equivalents of Malvidin 3,5-*O*-diGlc	3	6	3	19	12	11
Delphinidin 3-*O*-coumaroyl-Glc (*cis*- and *trans*-isomers)	NA	NA	As equivalents of Malvidin 3-*O*-Glc	5	6	4	13	14	13
Cyanidin 3-*O*-coumaroyl-Glc (*cis*- and *trans*-isomers)	NA	NA	As equivalents of Malvidin 3-*O*-Glc	7	7	7	24	29	12
Petunidin 3-O-coumaroyl-Glc (*cis*- and *trans*-isomers)	NA	NA	As equivalents of Malvidin 3-*O*-Glc	4	12	9	24	17	13
Peonidin 3-*O*-coumaroyl-Glc(*cis*- and *trans*-isomers)	NA	NA	As equivalents of Malvidin 3-*O*-Glc	3	10	4	15	29	30
Malvidin 3-*O*-coumaroyl-Glc(*cis*- and *trans*-isomers)	NA	NA	As equivalents of Malvidin 3-*O*-Glc	3	7	5	15	24	24
Delphinidin 3-*O*-coumaroyl-Glc-5-Glc (*cis*- and *trans*-isomers)	NA	NA	As equivalents of Malvidin 3,5-*O*-diGlc	3	9	3	18	16	18
Cyanidin 3-*O*-coumaroyl-Glc-5-Glc (*cis*- and *trans*-isomers)	NA	NA	As equivalents of Malvidin 3,5-*O*-diGlc	4	7	11	14	9	8
Petunidin 3-*O*-coumaroyl-Glc-5-Glc (*cis*- and *trans*-isomers)	NA	NA	As equivalents of Malvidin 3,5-*O*-diGlc	3	9	3	19	11	11
Peonidin 3-*O*-coumaroyl-Glc-5-Glc (*cis*- and *trans*-isomers)	NA	NA	As equivalents of Malvidin 3,5-*O*-diGlc	2	6	3	18	14	13
Malvidin 3-*O*-coumaroyl-Glc-5-Glc (*cis*- and *trans*-isomers)	NA	NA	As equivalents of Malvidin 3,5-*O*-diGlc	1	4	2	18	14	16
(epi)cat ^b^-ethyl-malvidin 3-*O*-Glc (4 isomers)	NA	NA	As equivalents of Malvidin 3-*O*-Glc	3	9	8	16	19	29
(epi)cat-ethyl-malvidin 3-*O*-acetyl-Glc (2 isomers)	NA	NA	As equivalents of Malvidin 3-*O*-Glc	4	8	6	14	12	13
(epi)cat-ethyl-malvidin 3-*O*-coumaroyl-Glc (2 isomers)	NA	NA	As equivalents of Malvidin 3-*O*-Glc	3	7	8	14	13	10
Pyranopeonidin 3-*O*-Glc	NA	NA	As equivalents of Malvidin 3-*O*-Glc	11	5	5	35	28	28
Pyranopetunidin 3-*O*-Glc	NA	NA	As equivalents of Malvidin 3-*O*-Glc	nd	nd	nd	nd	nd	nd
Pyranomalvidin 3-*O*-Glc (vitisin B)	NA	NA	As equivalents of Malvidin 3-*O*-Glc	12	18	9	19	17	14
Pyranomalvidin 3-*O*-acetyl-Glc	NA	NA	As equivalents of Malvidin 3-*O*-Glc	17	22	12	20	33	15
Pyranomalvidin 3-*O*-coumaroyl-Glc	NA	NA	As equivalents of Malvidin 3-*O*-Glc	20	14	5	26	27	13
Carboxypyranomalvidin 3-*O*-Glc (vitisin A)	NA	NA	As equivalents of Malvidin 3-*O*-Glc	6	6	3	14	12	11
Carboxypyranomalvidin 3-*O*-acetyl-Glc	NA	NA	As equivalents of Malvidin 3-*O*-Glc	7	8	6	14	11	13
Carboxypyranomalvidin 3-*O*-coumaroyl-Glc	NA	NA	As equivalents of Malvidin 3-*O*-Glc	8	6	6	18	11	14
Petunidin 3-*O*-caffeoyl Glc	NA	NA	As equivalents of Malvidin 3-*O*-Glc	13	6	5	17	15	17
Peonidin 3-*O*-caffeoyl Glc	NA	NA	As equivalents of Malvidin 3-*O*-Glc	4	11	7	24	19	12
Malvidin 3-*O*-caffeoyl Glc	NA	NA	As equivalents of Malvidin 3-*O*-Glc	5	11	5	18	17	12

^a^ Glc: glucoside; ^b^ epi(cat): catechin or epicatechin; ^c^ No standards available; ^d^ Not detected in wine sample. NA: not assessed.

## Data Availability

The raw data supporting the conclusions of this article will be made available by the authors on request.

## Supplementary Materials

The following supporting information can be downloaded at https://www.mdpi.com/article/10.3390/mps7050082/s1, Table S1: Accuracy values expressed in percentages for the five concentration levels of the calibration curves of the compounds; Table S2: Comparison of LODs between the method currently presented (Method 1) and the MRM method developed by Myrtsi et al. [7] (Method 2) for the shared compounds; Table S3: Comparison of linearity ranges between the method currently presented (Method 1), the MRM method developed by Myrtsi et al. [7] (Method 2), and the method developed by Lambert et al. [8] (Method 3) for the shared compounds; Figure S1: Total ion chromatogram of a wine sample used for repeatability calculation.
