# Supplementary material for: A Versatile Ultra-High-Performance Liquid Chromatography-Full-Scan High-Resolution Mass Spectrometry Method to Quantify Wine Polyphenols
Source: Methods Protoc. 2024 Oct 10;7(5):82. doi: 10.3390/mps7050082 (PMC11510656; doi:10.3390/mps7050082)

Table S1. Accuracy values expressed in percentages (*i.e.* % recovery) for the five concentration levels of the calibration curves.

| **Compounds** | **Concentration levels (μmol/L)** | | | | |
|---|---|---|---|---|---|
| | **250** | **50** | **10** | **5** | **1** |
| *Gallic acid* | 99 | 112 | 108 | 121 | 143 |
| *Protocatechuic acid* | 100 | 105 | 89 | 106 | 104 |
| *trans - caftaric acid* | 103 | 109 | 120 | 113 | 72 |
| *Catechin* | 100 | 91 | 102 | 85 | 85 |
| *Vanillic acid* | 101 | 81 | 104 | 94 | |
| *Procyanidin B1* | 100 | 97 | 103 | 103 | 169 |
| *Caffeic acid* | 99 | 115 | 105 | 114 | 106 |
| *Procyanidin B2* | 100 | 96 | 94 | 86 | 78 |
| *Epicatechin* | 100 | 90 | 100 | 86 | 87 |
| *Syringic acid* | 100 | 103 | 94 | 100 | 93 |
| *p-coumaric acid* | 99 | 126 | 102 | 102 | 145 |
| *Malvidin 3,5-O-diGlc* | 100 | 110 | 97 | 92 | 80 |
| *Procyanidin C1* | 100 | 105 | 95 | 85 | 70 |
| *Ferulic acid* | 100 | 101 | 78 | 81 | 130 |
| *trans - piceid* | | 99 | 127 | 114 | 111 |
| *Malvidin 3-O-Glc* | 99 | 119 | 112 | 106 | 94 |
| *Ethyl protocatechuate* | 100 | 102 | 91 | 96 | 83 |
| *Procyanidin A2* | 100 | 109 | 101 | 95 | 90 |
| *Quercetin 3-O-Glc* | 100 | 102 | 88 | 100 | 81 |
| *trans - resveratrol* | 100 | 88 | 94 | 80 | 70 |
| *Ethyl caffeate* | 100 | 106 | 97 | 104 | 102 |
| *Quercetin* | | 101 | 86 | 97 | 63 |
| *(+)-ε-Viniferin* | 99 | 113 | 117 | 100 | 94 |

Table S2. Comparison of LOD between the method currently presented (Method 1), the MRM method developed by Myrtsi et al [7] (Method 2) for the shared compounds.

| Compound | Method 1 LOD (mg/L) | Method 2 LOD (mg/L) |
|---|---|---|
| *Gallic acid* | 0.003 | 0.029 |
| *Protocatechuic acid* | 0.003 | 0.029 |
| *trans - caftaric acid* | 0.003 | 0.025 |
| *Procyanidin B1* | 0.004 | 0.053 |
| *Catechin* | 0.011 | 0.094 |
| *Procyanidin B2* | 0.011 | 0.06 |
| *Epicatechin* | 0.012 | 0.046 |
| *Caffeic acid* | 0.011 | 0.012 |
| *Syringic acid* | 0.003 | 0.119 |
| *Quercetin 3-O-Glc* | 0.006 | 0.041 |
| *p-coumaric acid* | 0.023 | 0.030 |
| *trans - resveratrol* | 0.011 | 0.158 |
| *Quercetin* | 0.024 | 0.031 |
| *Malvidin 3-O-Glc* | 0.040 | 0.020 |

Table S3. Comparison of linearity range between the method currently presented (Method 1), the MRM method developed by Myrtsi et al [7] (Method 2) and the method developed by Lambert et al [8] (Method 3) for the shared compounds.

| Compound | Method 1 (mg/L) | Method 2 (mg/L) | Method 3 (mg/L) |
|---|---|---|---|
| *Gallic acid* | 0.17 - 42.5 | 0.01 - 90 | 0.05 - 1.5 |
| *Protocatechuic acid* | 0.15 - 38.5 | 0.01 - 20 | 0.05 - 1.5 |
| *trans - caftaric acid* | 0.31 - 78.1 | 0.01 - 100 | 0.05 - 1.5 |
| *Procyanidin B1* | 0.58 - 144.1 | n.a | 0.05 - 1.5 |
| *Catechin* | 0.29 - 72.6 | 0.01 - 50 | 0.05 - 1.5 |
| *Procyanidin B2* | 0.58 - 144.1 | 0.01 - 15 | 0.05 - 1.5 |
| *Epicatechin* | 0.29 - 72.6 | 0.01 - 50 | 0.05 - 1.5 |
| *Caffeic acid* | 0.18 - 45.0 | 0.01 - 50 | 0.05 - 1.5 |
| *Syringic acid* | 0.20 - 49.5 | 0.01 - 20 | 0.05 - 1.5 |
| *Quercetin 3-O-Glc* | 0.46 - 116.1 | 0.01 - 30 | 0.05 - 1.5 |
| *p-coumaric acid* | 0.16 - 41.0 | 0.01 - 20 | 0.05 - 1.5 |
| *trans - resveratrol* | 0.23 - 57.1 | 0.01 - 20 | 0.05 - 1.5 |
| *Quercetin* | 0.30 - 75.6 | 0.01 - 10 | 0.05 - 1.5 |
| *Malvidin 3-O-Glc* | 0.49 - 123.4 | 0.01 - 50 | 0.05 - 1.5 |

Figure S1. Total ion chromatogram of a wine sample used for repeatability calculation.

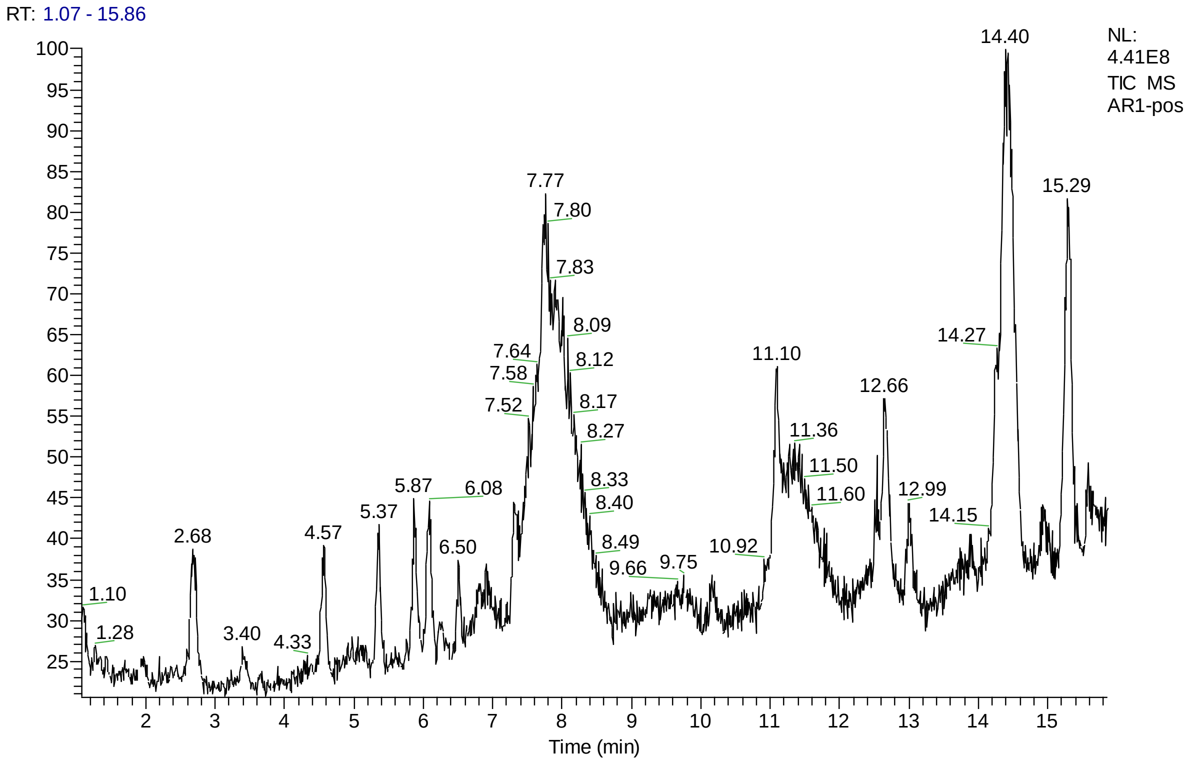

Supplement: Supplementary file 1 [file mps-07-00082-s001.zip › mps-3136233-supplementary.pdf]
